# Supplementary material for: From sequence to enzyme mechanism using multi-label machine learning
Source: BMC Bioinformatics. 2014 May 19;15:150. doi: 10.1186/1471-2105-15-150 (PMC4229970; doi:10.1186/1471-2105-15-150)
Supplement: Additional file 2 — Java code of ml2db. Additional file ml2db_code.tar.gz contains the Java source code to run the multi-label machine learning experiments and save the results to database. The code’s Javadoc is included. [file 1471-2105-15-150-S2.zip › additional file 2/ml2db/ml2db/doc/mulan/classifier/lazy/package-summary.html]

mulan.classifier.lazy


JavaScript is disabled on your browser.


- Overview
- Package
- Class
- Use
- Tree
- Deprecated
- Index
- Help

- Prev Package
- Next Package

- Frames
- No Frames

- All Classes

# Package mulan.classifier.lazy

- Class Summary

  | Class | Description |
  | --- | --- |
  |  |  |
  | --- | --- |
  | BRkNN | Simple BR implementation of the KNN algorithm.For more information, see    Eleftherios Spyromitros, Grigorios Tsoumakas, Ioannis Vlahavas: An Empirical Study of Lazy Multilabel Classification Algorithms. |
  | IBLR\_ML | This class is an implementation of the "IBLR-ML" and "IBLR-ML+" methods for the MULAN package.    For more information, see    Weiwei Cheng, Eyke Hullermeier (2009). |
  | MLkNN | Class implementing the ML-kNN (Multi-Label k Nearest Neighbours) algorithm.    For more information, see    Min-Ling Zhang, Zhi-Hua Zhou (2007). |
  | MultiLabelKNN | Superclass of all KNN based multi-label algorithms |
- Enum Summary

  | Enum | Description |
  | --- | --- |
  |  |  |
  | --- | --- |
  | BRkNN.ExtensionType | The two types of extensions |

- Overview
- Package
- Class
- Use
- Tree
- Deprecated
- Index
- Help

- Prev Package
- Next Package

- Frames
- No Frames

- All Classes
